# Co-overexpression of native phospholipid-biosynthetic genes *plsX* and *plsC* enhances lipid production in *Synechocystis* sp. PCC 6803

**DOI:** 10.1038/s41598-018-31789-5

**Published:** 2018-09-10

**Authors:** Umaporn Towijit, Nutchaya Songruk, Peter Lindblad, Aran Incharoensakdi, Saowarath Jantaro

**Affiliations:** 1 Laboratory of Cyanobacterial Biotechnology, Department of Biochemistry, Faculty of Science, Chulalongkorn University, Bangkok, 10330 Thailand; 2 Program of Biotechnology, Faculty of Science, Chulalongkorn University, Bangkok, 10330 Thailand; 3 Microbial Chemistry, Department of Chemistry – Ångström, Uppsala University, Box 523, SE-75120 Uppsala, Sweden

## Abstract

The overexpression of native *plsX* and *plsC* genes involving in fatty acid/phospholipid synthesis first timely-reported the significantly enhanced lipid contents in *Synechocystis* sp. PCC 6803. Growth rate, intracellular pigment contents including chlorophyll *a* and carotenoids, and oxygen evolution rate of all overexpressing (OX) strains were normally similar as wild type. For fatty acid compositions, saturated fatty acid, in particular palmitic acid (16:0) was dominantly increased in OX strains whereas slight increases of unsaturated fatty acids were observed, specifically linoleic acid (18:2) and alpha-linolenic acid (18:3). The *plsC/plsX*-overexpressing (OX + XC) strain produced high lipid content of about 24.3%w/dcw under normal condition and was further enhanced up to 39.1%w/dcw by acetate induction. This OX + XC engineered strain was capable of decreasing *phaA* transcript level which related to poly-3-hydroxybutyrate (PHB) synthesis under acetate treatment. Moreover, the expression level of gene transcripts revealed that the *plsX*- and *plsC/plsX*-overexpression strains had also increased *accA* transcript amounts which involved in the irreversible carboxylation of acetyl-CoA to malonyl-CoA. Altogether, these overexpressing strains significantly augmented higher lipid contents when compared to wild type by partly overcoming the limitation of lipid production.

## Introduction

The third generation of renewable energy resource, especially cyanobacteria, does not compete with human food resources when compared to first and second generations^1^. The engineering of biochemical pathways in cyanobacteria involved in specific energy types opens up possibilities to develop sustainability of energy resource. Cyanobacteria are photosynthetic prokaryotes which may effectively convert solar energy and carbon dioxide into chemicals and biofuels. Energy storage components in biological system are mainly lipids, carbohydrates, proteins^1^ which can be used to produce various fuels like ethanol^2^, alka(e)nes^3^, oil and biodiesel^4^, 1-butanol^5^, fatty alcohol^6^, hydrogen^7,8^. In order to synthesize more lipid energy storage components, the integration between metabolic engineering and system biology is a common approach^9,10^. The main biological functions of lipids include energy storage, structural components of cell membranes, and important signaling molecules. Fatty acids are parts of lipids which are synthesized by chain-elongation of an acetyl Co-A primer with malonyl Co-A or methylmalonyl Co-A groups in a process called fatty acid synthesis.

**Figure 1:**
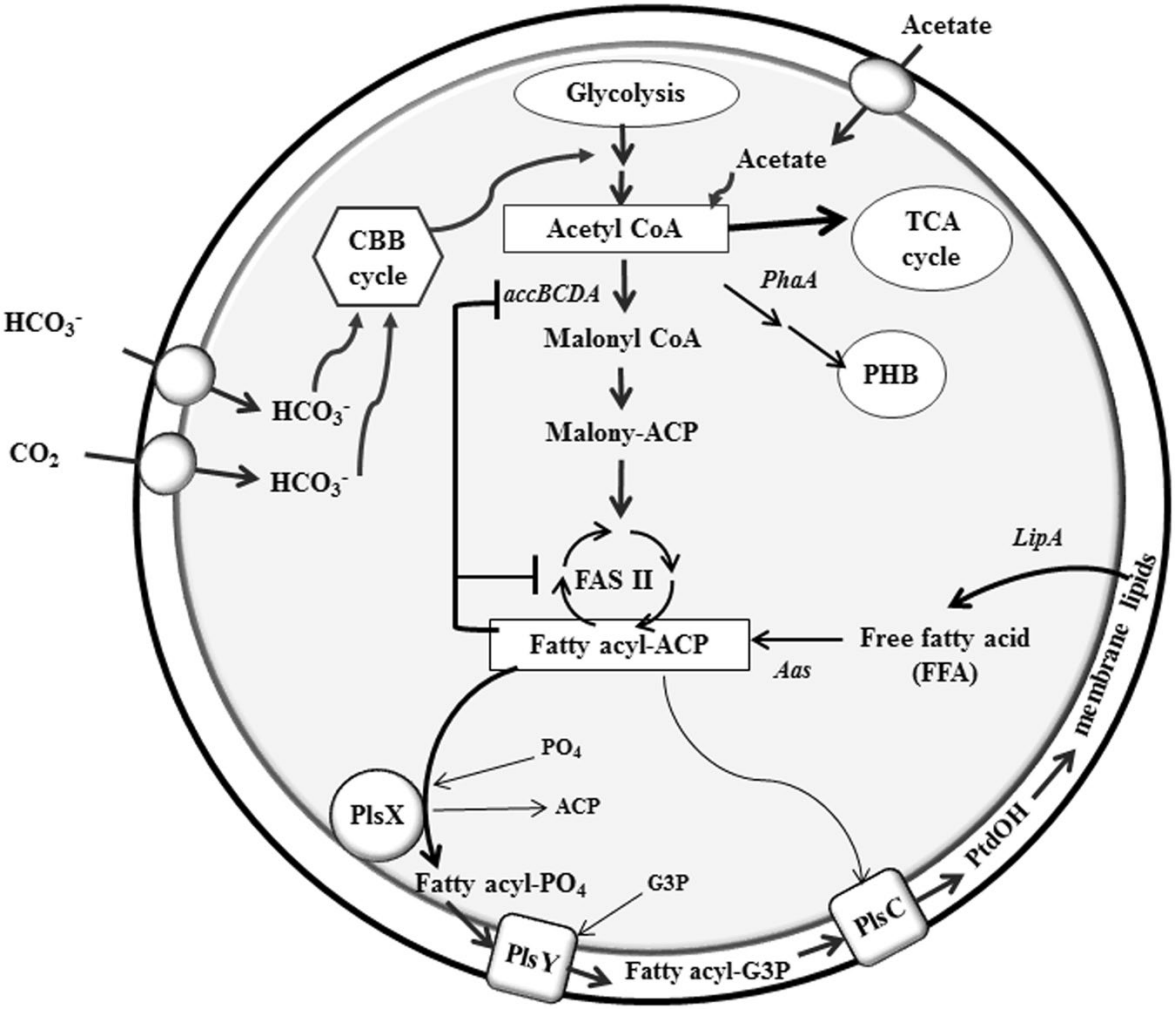
Overview of metabolic pathways representing the conversion of acetyl-CoA to membrane lipid biosynthesis under growth condition in the unicellular cyanobacterium *Synechocystis* sp. PCC 6803 (modified from^1^ and^14^). Abbreviations AAS: putative acyl-ACP synthetase, *acc*BCDA: Acetyl-CoA carboxylase gene subunits BCDA, ACP: Acyl Carrier Protein, CBB: Calvin-Benson-Bassham, G3P: Glyceraldehyde-3-phasphate, LipA: putative lipase, PhaA: beta-ketothiolase, PHB: poly-3-hydroxybutyrate, PlsX: fatty acid/phospholipid synthesis protein or putative phosphate acyltransferase, PlsY: putative acylglycerol-P acyltransferase (no data available in Cyanobase), PlsC: putative 1-acyl-glycerol-P acyltransferase, PtdOH: phosphatidic acid and TCA: Tricarboxylic acid.

In the cyanobacterium *Synechocystis* sp. PCC 6803, the main intermediate for fatty acid and phospholipid, or membrane lipid, metabolism (Fig. 1) is acetyl Co-A from the glycolysis pathway. Acetyl Co-A flux is directly fed into pathways such as the TCA cycle, PHB biosynthesis, glycogen biosynthesis and fatty acid biosynthesis. Acetyl Co-A is converted to malonyl-CoA in a rate-limiting reaction catalysed by a multi-subunit acetyl-CoA carboxylase consisting of AccA (encoded by *slr0728*). First, the malonyl subunit from malonyl-CoA is transferred to ACP by the malonyl-CoA:ACP transacylase (FabD, EC 2.3.1.39). The resulting malonyl-ACP is then condensed to acetyl CoA with the activity of 3-ketoacyl-ACP synthase (FabH, EC 2.3.1.41). The formed fatty acyl ACPs are later directed to the synthesis of membrane glycerolipids. The enzyme lysophosphatidic acid acyltransferase or 1-sn-glycerol-3-phosphate acyltransferase (LPAAT; PlsC; EC 2.3.2.51) catalyzes the second step in phospholipid biosynthesis, and its function might close proximity to the first step catalyzed by glycerol-3-phosphate acyltransferase (GPAT). This enzyme can be utilize either acyl-Coenzyme A or acyl-acyl carrier protein as the fatty acyl donor at sn-2 position. Nowadays, the LPAAT of *Synechocystis* sp. PCC 6803 has already been identified as *sll1752* and *sll1848* (*plsC*)^11,12^ while the gene encoded GPAT has not yet been identified. Okazaki and co-workers^12^ reported that disruption of *sll1848* (Δ*sll1848*) dramatically decreased the relative levels of palmitic acids (16:0) at the sn-2 position which is replaced by C18 acids. Moreover, the product of *sll1848* overexpressed in *E. coli* had 130-fold higher specific activity, as LPAAT, for 16:0-CoA than for 18:0-CoA when examined with acyl-CoAs as substrate instead of acyl-ACPs. These results indicated that *sll1848* encodes the major LPAAT, which has strong specificity for 16:0-ACP. Another LPAAT, *sll1752* in *Synechocystis*, is a minor LPAAT that its activity prefer 18:0-CoA rather than 16:0-CoA^12^. The chloroplast LPAAT that is encoded by the ATS2 gene of a higher plant *Arabidopsis thaliana*^13^ is structurally similar to the product of *sll1848*. In microorganisms, the fatty acyl-ACP is directly added into a PG molecule (backbone for the glycerolipid synthesis) by a sn-glycerol-3-phosphate acyl-transferase (EC 2.3.1.15, GPAT or PlsB) or by a newly discovered two-reaction system catalyzed by the enzymes, PlsX and PlsY^14^. Phosphate is added into the fatty acyl group derived from a fatty acyl-ACP chain by PlsX catalyzing and then transferred into G-3-P molecule catalyzed by PlsY^15^. In *Bacillus subtilis*, the roles of *plsX*, *plsY* and *plsC* were investigated^16^. Long-chain acyl-ACPs are the end products of the bacterial dissociated type II fatty acid synthase system (FAS II). *B. subtilis* uses PlsX to convert acyl-ACPs to acyl-PO_4_ via a phosphotransacylase-type reversible reaction. The next step is catalyzed by the membrane-associated PlsY (acylglycerol-P acyltransferase) encoded by *yneS* that transfers the acyl moiety to the 1 position of glycerol-P to form acyl-G3P. Acylation of the 2 position is catalyzed by PlsC (YhdO), a membrane-bound 1-acyl-glycerol-P acyltransferase that specifically uses acyl-ACP as the acyl donor to form PtdOH. The functions of those three genes were studied by the constructions of knockout strains of three genes in *B. subtilis*. The *plsX*-depleted cells were inactivated on fatty acid and phospholipid systems. Thus *B. subtilis* mutant could not produce long chain acyl-ACP end product of fatty acid synthesis. On the other hand, *plsY*-depleted cells also blocked phospholipid synthesis whereas *plsC*-depleted cells accumulated monoacylglycerol with a high amount of fatty acid. PlsC catalyzed the transfer of fatty acid to the 2-position of Acyl-G3P via acyl-ACP into phosphatidic acid (PtdOH), the key intermediate of phospholipid synthesis. On the other hand, the membrane phospholipids are degraded by lipase A (encoded by *lipA*) into free fatty acids, and could further pass through a recycling process to fatty acyl-ACP via acyl-ACP synthetase (encoded by *aas*) (Fig. 1). In this study, we constructed three engineered *Synechocystis* 6803 strains overexpressing *plsX*-, *plsC*- and *plsC/plsX* which significantly enhanced lipid production compared to *Synechocystis* PCC 6803 wild type.

## Results

### Overexpression of native PlsX and PlsC in cells of Synechocystis sp. PCC 6803

**Figure 2:**
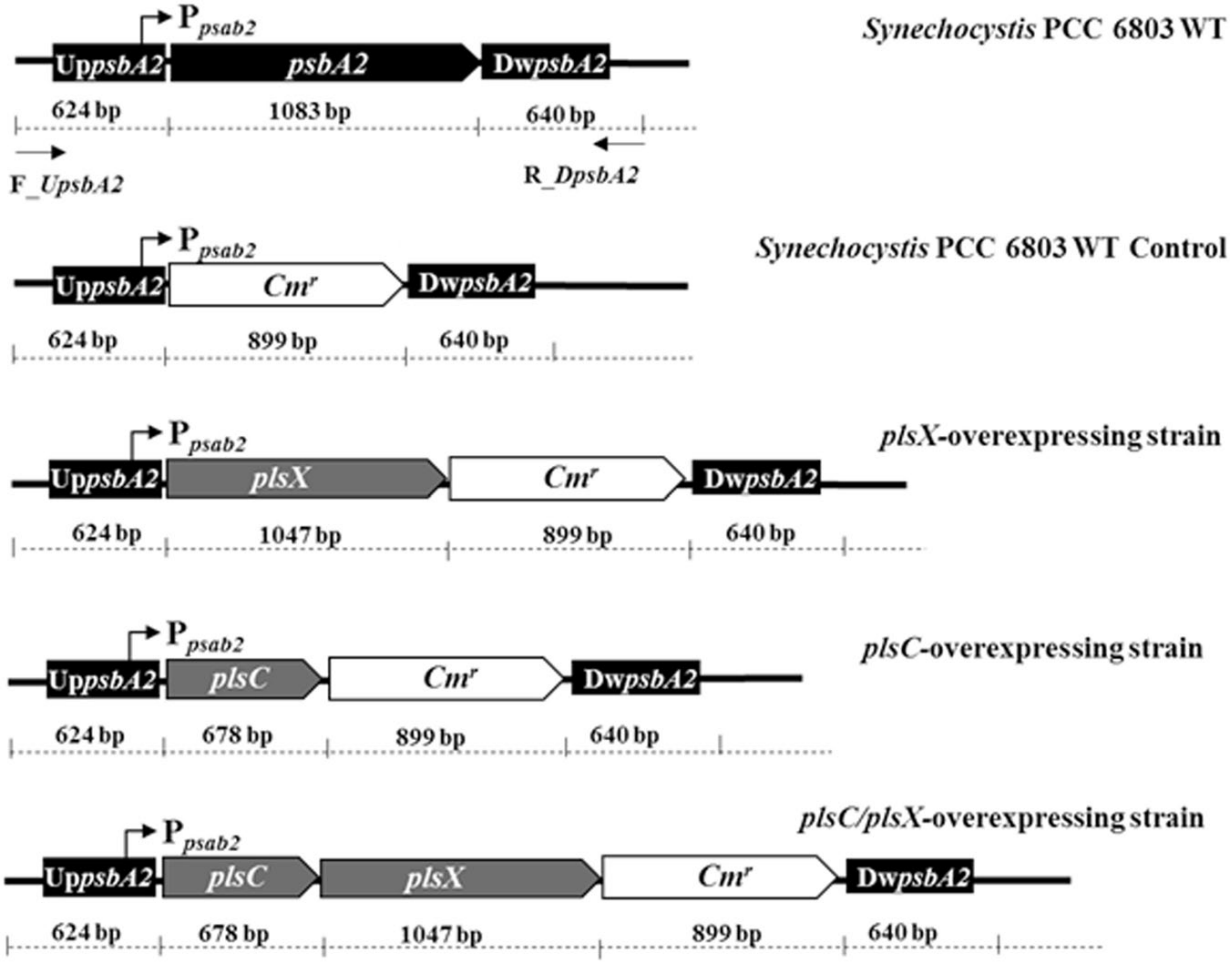
Physical map representing *psbA2* locus in *Synechocystis* sp. PCC 6803 wild type (WT), with the inserted *plsX, plsC* and *plsC/plsX* genes in different engineered strains, hereinafter OX + X, OX + C and OX + XC, respectively. The specific primers (Table 2) were used to recombine each gene into *Synechocystis* genome. The WT control cells contained an inserted *Cm*^*r*^ gene cassette in their genome. The size of each gene fragment was shown correspondingly under the map.

**Figure 3:**
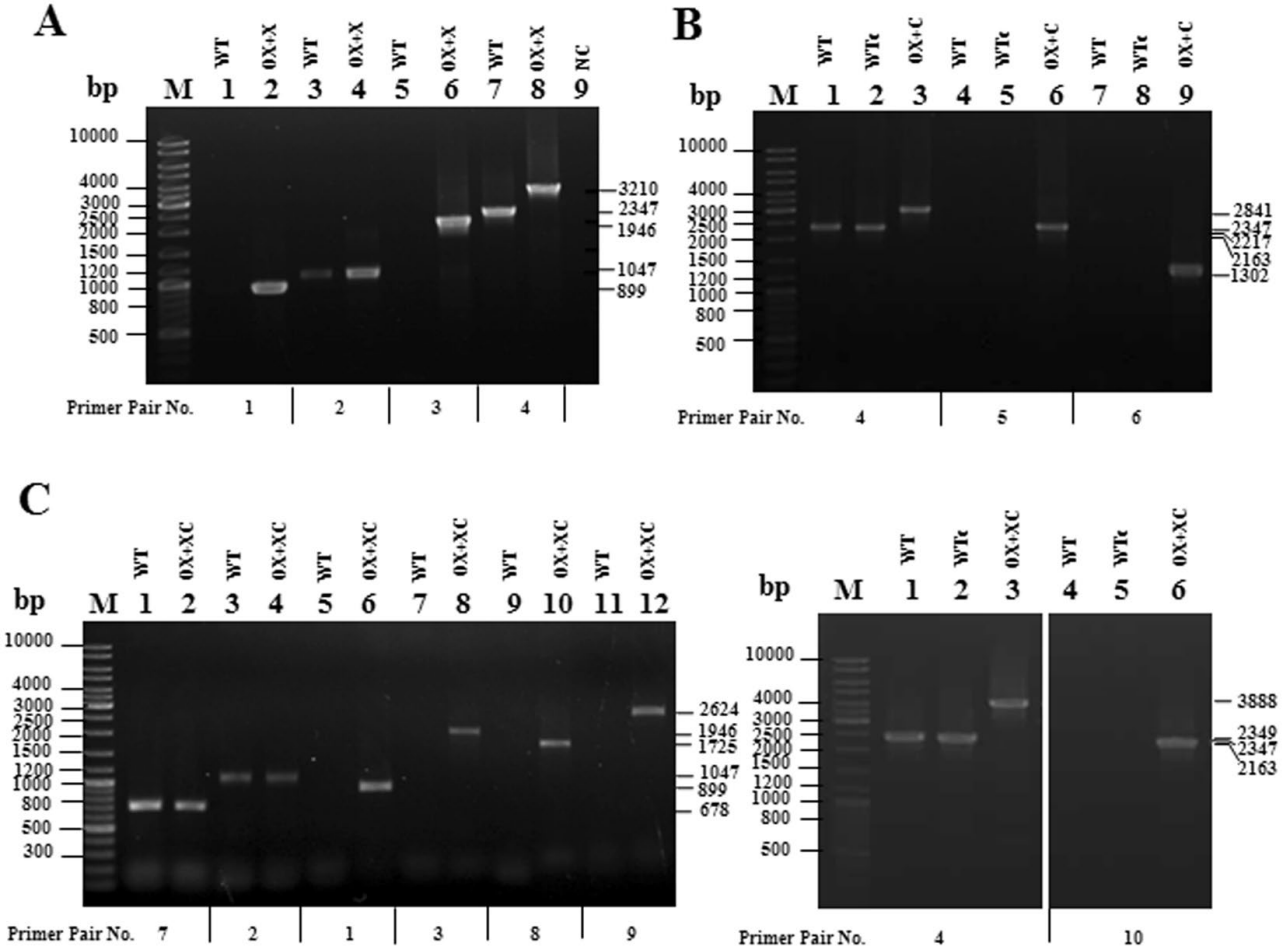
Confirmation of insertion and complete segregation using PCRs with genomic DNA as the template from WT, WTc and the OX strains, including OX + X (**A**), OX + C (**B**) and OX + XC (**C**), corresponding to the physical map in Fig. 2. Lane M: GeneRuler^TM^ DNA ladder (Fermentas). The different primer pairs was used to amplify each gene fragment as indicated in Table 3 including different 10 pairs of primers. The cropped gels (in C) were taken from the same gel cutting out the repeated bands of transformants as shown in Supplementary information.

**Figure 4:**
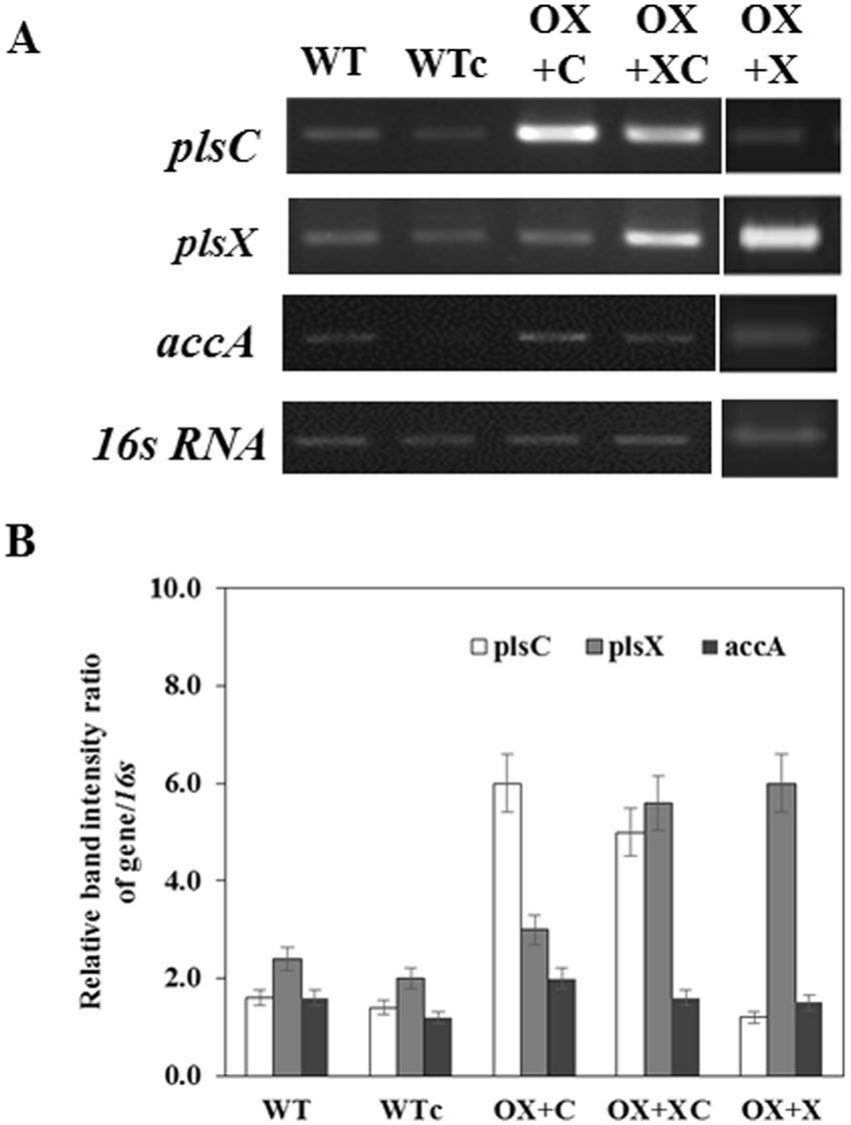
Relative transcript levels of *plsC, plsX and accA* performed by RT-PCR in WT, WTc and OX strains grown under normal growth condition (**A**). The *16 s* RNA was used as reference control. The ratios of relative band intensity of gene/*16 s* were shown in mean ± S.D. (n = 3) (**B**). The cropped gels of OX + X were taken from the different gels as shown in Supplementary information.

To prove our hypothesis whether *Pls* gene overexpressions could enhance the intracellular lipids, the expression vector pEERM^17^ was inserted separately by each native gene of *plsX* or *plsC* or co-inserted by both *plsC/plsX* genes (Fig. 2). The *Synechocystis* WT control (WTc) was WT containing empty pEERM vector with resistant cassette. The obtained constructs were transformed into the *Synechocystis* genome through homologous recombination via flanking regions of *psbA2* gene. Expected transformants of all constructed recombinants were successfully obtained including OX + X, OX + C and OX + XC. The complete segregation of each transformant and correct gene localization in *Synechocystis* genome were confirmed by PCR using different pairs of primers (Fig. 3 and Table 1). For each strain, we also confirmed the transcription of the introduced genes using RT-PCR (Fig. 4). Increased transcript levels were observed for either *plsC* or *plsX* in OX + C or OX + X, respectively, whereas *plsC*/*pls*X co-overexpression was enhanced both in relative amount. A slight increase of *accA* transcript, encoding acetyl Co-A carboxylase, was noted for the OX + C strain when compared to those of WT and WTc. The result also revealed that the *plsC*-overexpressing strain, namely OX + C, enabled to induce *plsX* transcript level but not vice versa.

**Table 1:** Pairs of primers used for the confirmation of insertion and complete segregation in wild type (WT) and its transformants.

Pair No.	Primers			Expected size of gene fragment (bp)			
	Forward	Reverse	WT	WTc	OX + X	OX + C	OX + XC
1	*Cm* ^*r*^	*Cm* ^*r*^	—	899	899	899	899
2	*PlsX*	*PlsX*	1047	1047	1047	1047	1047
3	*PlsX*	*Cm* ^*r*^	—	—	1946	—	1946
4	*U_psbA2*	*D_psbA2*	2347	2163	3210	2841	3888
5	*PlsC*	*D_psbA2*	—	—	—	2217	3264
6	*U_psbA2*	*PlsC*	—	—	—	1302	1302
7	*PlsC*	*PlsC*	678	678	678	678	678
8	*PlsC*	*PlsX*	—	—	—	—	1725
9	*PlsC*	*Cm* ^*r*^	—	—	—	1577	2624
10	*U_psbA2*	*PlsX*	—	—	1671	—	2349

Note: “—” Means no band of gene fragment.

**Figure 5:**
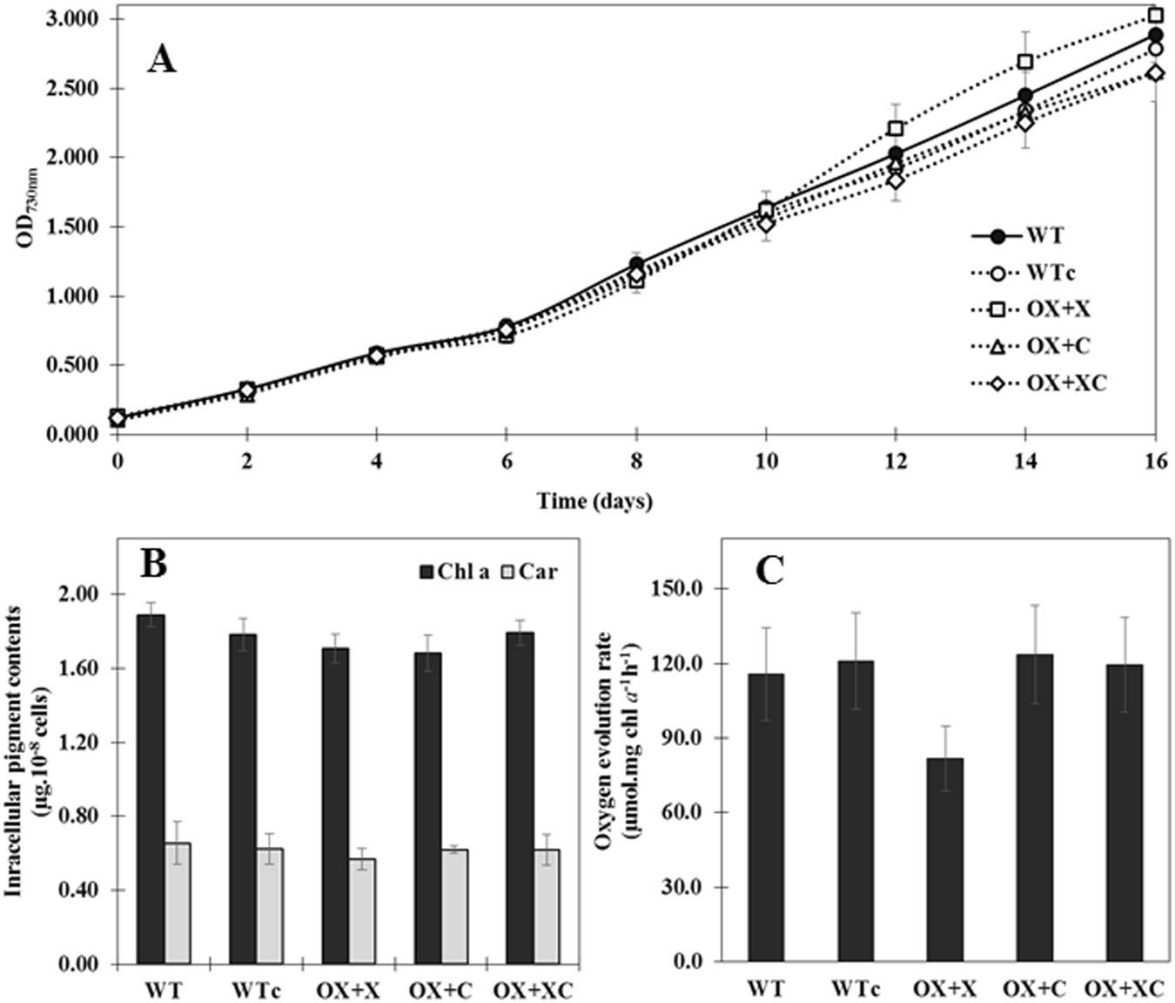
The optical density (OD) at 730 nm (**A**) of 16 day-cell culture, chlorophyll *a* (Chl a) and carotenoid (Car) contents (**B**) and oxygen evolution rate (**C**) of each strain grown at mid-log phase. Data represent mean ± S.D. (n = 3). Statistical significance between those levels of WT and OX strains was represented at *p* < 0.05.

Growth curves of all transformants showed the insignificant difference with either *Synechocystis* WT or WTc cells (Fig. 5A). WTc cells contained the antibiotic cassette in empty vector were enabled to grow as similar as WT, as well as their intracellular pigment levels and oxygen evolution rate (Fig. 5B,C). Only OX + X and OX + C strains gave significantly lower amounts of chlorophyll *a* when compared to WT (Fig. 5B) whereas there were no differences in carotenoid contents (Fig. 5C). The oxygen evolution rates, represented as photosynthetic efficiency, were in similar level in all strains ranging from 115–123 μmol O_2_ mg Chl *a*^−1^h^−1^, except OX + X which gave the lowest oxygen evolution rate of about 81.7 μmol O_2_ mg Chl *a*^−1^h^−1^ (Fig. 5C). It was interesting that strain OX + C with its lower chlorophyll *a* content had a higher oxygen evolution rate compared to other strains.

### Lipid production in all overexpression strains

**Figure 6:**
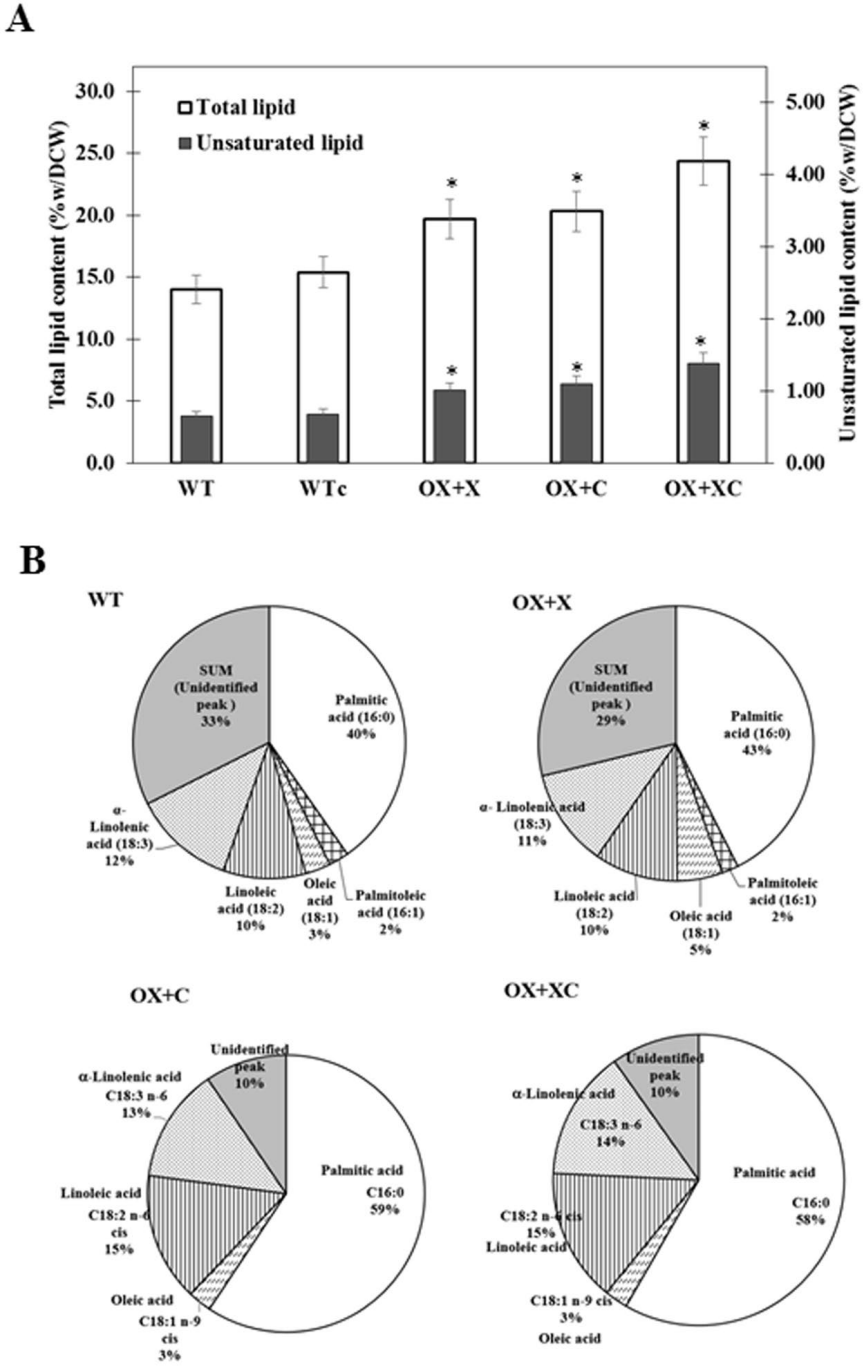
The contents of total lipid and unsaturated lipid (**A**) and the fatty acid compositions measured by GC instrument (**B**) of WT, WTc, and OX strains. Data represent mean ± S.D. (n = 3). Statistical significance between those levels of WT and OX strains was represented at *p* < 0.05.

In order to compare the consequence of our metabolic engineering-designed strains, we determined their lipid products including total lipids, total unsaturated lipids and fatty acid composition. After we determined the effect of growth phase on lipid production, the log phase-growing WT cells gave the highest amounts of total lipids (data not shown). The OX + X, OX + C and OX + XC overexpressing strains obviously produced higher contents of total lipids than that of WT (about 14.0%w/dcw) at mid-log phase of growth under normal growth condition, 19.7, 20.3 and 24.3%w/dcw, respectively (Fig. 6A). Likewise, a slight induction of total unsaturated fatty acids was noted, 1.0, 1.1 and 1.4%w/dcw, respectively, when compared to that of WT with 0.7%w/dcw (Fig. 6A). The fatty acid compositions of each strain was changed from WT (Fig. 6B), as well as an apparent decrease of unidentified lipid was shown in OX strains. The significant enhancement of palmitic acid composition, a saturated fatty acid, was in a range of about 43–59% when compared to that of WT at 40%, as well as the unsaturated fatty acid compositions were increased from 27% to 28%, 31% and 32% in OX + X, OX + C and OX + XC, respectively. On the other hand, the OX + C and OX + XC strains were apparently induced changes in unsaturated fatty acids including linoleic acid (18:2 or omega-6) increased from 10% to 15% and α-linolenic acid (18:3 or omega-3) from 12% to 13% and 14% respective increases.

### Effect of acetate supplementation on lipid production

**Figure 7:**
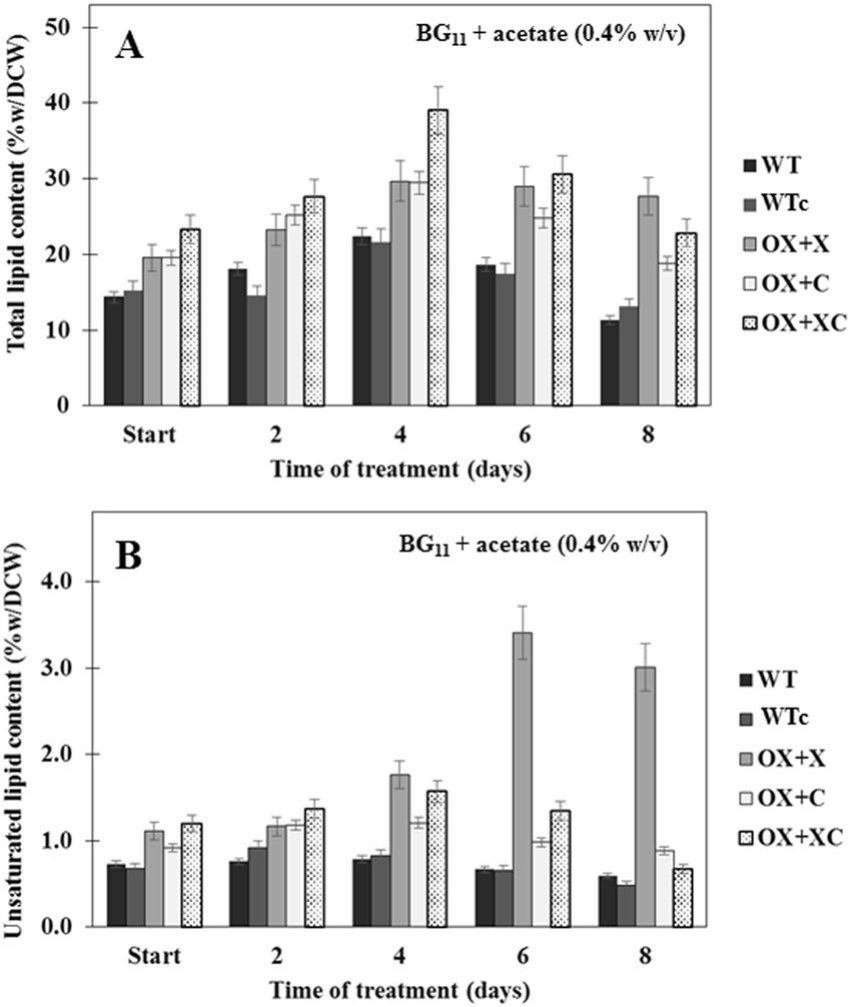
Effect of acetate supplementation on contents of total lipid (**A**) and unsaturated lipid (**B**) in WT, WTc, and OX strains at time indicated. Data represent mean ± S.D. (n = 3).

Prompted by the previous report that the carbon source supplementation, in particular acetate, enhanced the acetyl-CoA production and PHB content^18^, we then treated whether the acetate addition was also induced total lipids up from normal growth culture. As shown in Fig. 7, the acetate supplemented cultures (0.4%w/v) were treated for 8 days after mid-log phase. At start treatment, the total lipid and unsaturated lipid contents were from mid-log phase cells of all strains. After acetate treatment for 4 days, the highest amount of total lipids was shown in OX + XC strain with about 39.1%w/dcw when compared to other strains (Fig. 7A). Actually, each single overexpression of OX + X and OX + C was also increased on their total lipid content of about 29.6 and 29.5%w/dcw, respectively, as well as WT, increased its total lipid content up to 22.4%w/dcw. On the other hand, contents of unsaturated fatty acids of OX strains, in particular OX + X and OX + XC, were noticed at day 4 of treatment whereas the sharply increased amounts of unsaturated fatty acids were shown at day 6 and day 8 of acetate treatment up to about 3.4 and 3.0%w/dcw, respectively (Fig. 7B).

**Figure 8:**
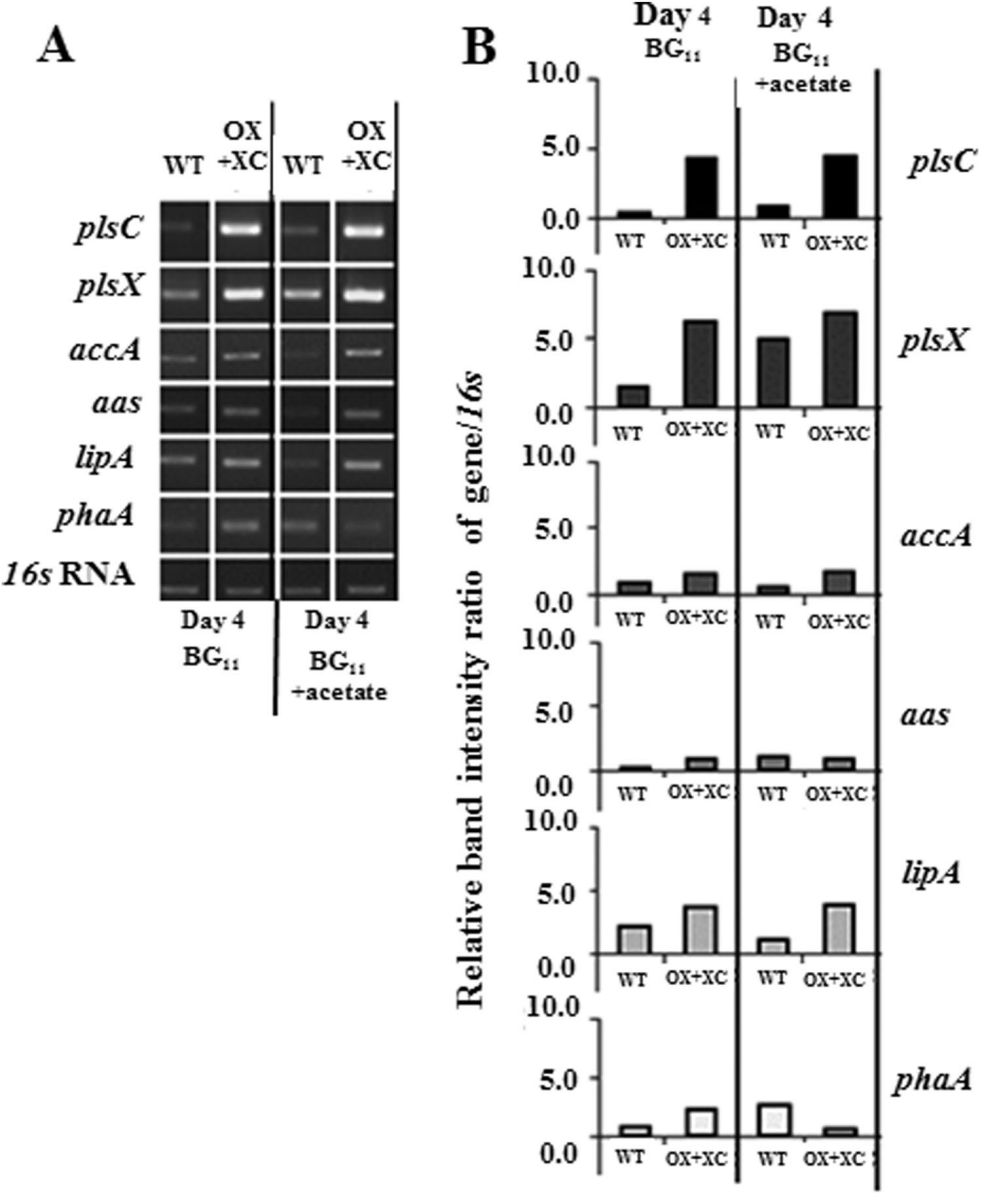
Relative transcript levels of *plsC, plsX, accA, aas, lipA* and *phaA* performed by RT-PCR in WT and OX + XC strain when cells supplemented with acetate at day 4 of treatment (**A**). The *16s* RNA was used as reference control. The ratios of relative band intensity of gene/*16s* were shown in (**B)**. The cropped gels of WT and OX + XC were taken from the different gels as shown in Supplementary information.

To gain more understanding of metabolic flow in term of transcription level, we also determined the expression of some genes involved in fatty acid and phospholipid metabolism. As shown in Fig. 8, the comparison between WT and OX + XC which gave highest lipid production at day 4 of treatment was determined. Under normal BG_11_ condition as control, OX + XC has increased transcript levels of not only *plsC* and *plsX* genes but also *accA* (acetyl Co-A carboxylase subunit A), *aas* (acyl-ACP synthetase), *lipA* (lipase A) and *phaA* (β-ketothiolase gene) compared to those of WT. It was surprising that the co-overexpression of *PlsC/PlsX* genes could induce *PhaA* transcript level, in a competing pathway that converts acetyl Co-A to PHB. When we treated cells with acetate for 4 days, WT cells was up-regulated on *plsX*, *plsC*, *aas* and *phaA* transcript levels whereas *accA* and *lipA* transcript amounst were decreased. For OX + XC strain, the *phaA* transcript level of OX + XC was obviously decreased by acetate induction.

## Discussions

In this current study, we first-timely report the enhanced lipid production by genetically modified *Synechocystis* sp. PCC 6803 strains with overexpressed genes involved in phospholipid metabolism (Fig. 1). In order to overcome the limitation of lipid production, we then constructed those engineered strains with overexpressing-*plsX*, -*plsC* and *PlsC/PlsX* genes which existed as down-stream protein/enzymes redirecting fatty acyl-ACP towards phospholipid biosynthesis.

In *Synechocystis* sp. PCC 6803, the sequence data of *PlsX* (*slr1510*) and *PlsC* (*sll1848*) are available from Cyanobase (http://genome.microbedb.jp/CyanoBase). We then analyzed the phylogenetic tree using the Neighbor-Joining method of *Synechocystis* 6803-PlsX and PlsC amino acid sequences compared to other cyanobacteria and out-group organisms (as shown in Figs S1 and S2, Tables S1 and S2 of Supplementary materials). *Synechocystis* PlsX amino acid sequence was identified as putative phosphaste acyltransferase whereas *Synechocystis* PlsC was identified as putative 1-acyl-sn-glycerol-3-phosphate acyltransferease. We demonstrated that *Synechocystis* PlsX and PlsC amino acid sequences had common ancestors with the acyltransferase amino acid sequence of other unicellular cyanobacteria such as *Synechococcus*. This phospholipid synthesis in *Synechocystis* is consistent with that in some bacteria such as *Bacillus subtilis* which has three genes related to biosynthetic pathway of membrane phospholipids including *plsX*, *plsY* and *plsC*^16^. The phylogenetic distribution of PlsX/PlsY/PlsC acyltransferase system for phospholipid synthesis was reported in cyanobacteria whereas some prokaryotes had both PlsB/PlsC and PlsX/PlsY/PlsC acyltransferase systems^14^. Recently in Cyanobase genome database, the sequence data of PlsY (acyl-phosphate:glycerol-3-phosphate O-acyltransferase) in some cyanobacteria were predicted such as D082_01810 gene ID of *Synechocystis* sp. PCC 6714, CWATWH8502_2705 gene ID of *Crocosphaera watsonii* WH 8502, O53_2732 gene ID of *Microcystis aeruginosa* TAIHU98, EV05_1151 gene ID of *Prochlorococcus* sp. MIT 0601, SynWH8103_00663 gene ID of *Synechococcus* sp. WH 8103, except *Synechocystis* sp. PCC 6803. However, the high identity of D082_01810 gene ID of *Synechocystis* sp. PCC 6714 was reported with *sll1973* (hypothetical protein) of *Synechocystis* sp. PCC 6803 for about 87.8%. Thus, it may be of interest further to construct cyanobacterial *plsXYC*-overexpressing strain, since there was recently reviewed that cyanobacteria had PlsX/PlsY in membrane lipids biosynthesis^1^ which was crucial component of energy storage in biological system.

We also demonstrated that our overexpression technique via the interruption of *psbA2* gene, in PEERM vector^17^, of all overexpression strains did not severely harm to their growth and photosynthetic characteristics. Previous studies reported that although the D1 protein of photosystem II in cyanobacteria is encoded by *psbA, which* includes *psbA1*, *psbA2* and *psbA3*, there is a compensatory expression of *psbA3* when *psbA2* is deleted with no phenotypic change being normally grown under growth condition^17,19,20^.

The overexpression of genes involved in phospholipid metabolism in this study, in particular *plsX* and *plsC*, in *Synechocystis* cells effectively increased their lipid production up to 24.3%w/dcw when compared to WT. It was interesting although the co-overexpressing strain of both *plsX* and *plsC* genes accumulated the highest amount of total lipids with almost 2 fold-higher than WT, it was about 1.2 fold-higher than single overexpression of either *plsX* or *plsC*. The achievable results of our study would partly overcome the limitation upon feedback inhibition mechanism of fatty acyl-ACP to acetyl-CoA carboxylase and FAS II, as our finding supported not only the increased lipid amount but also in the induced *accA* transcript of OX strain (Figs 4 and 8). On the other hand, the slight increases of unsaturated fatty acids, including omega-3 and omega-6, was apparently shown in *plsC*- and *plsC/plsX*-overexpressing strains. Not only biofuel resource, but cyanobacteria and algae are also beneficial resources of two polyunsaturated fatty acids (PUFAs), essential nutrients, including omega-3 and omega-6^21^. Normally, the ratio of saturated: unsaturated fatty acids in phospholipids potentially indicates the membrane fluidity which could be altered by temperature^22,23^. Previous reports in *Escherichia coli* revealed that cycles of elongation in FASII performed by FabB or FabF had crucial role in unsaturated fatty acid synthesis^24–26^. However, in this study, we found that the *plsC*- and *plsC/plsX*-overexpressing strains were capable of inducing the higher ratio production of unsaturated fatty acids. On the other hand, the promising activity data of acetyl Co-A carboxylase and Pls enzymes, with recently measured by radioactive or LC/MS/MS assays^27–29^, need to be quantified for further gaining more clarification on protein/enzyme level.

In this study, we showed that the enhancement of more lipid production was achieved by acetate supplementation (0.4%w/v), as shown about 1.7 fold higher than WT under the same day of treatment. Acetate is a crucial substrate for acetyl Co-A product, a hub intermediate for main TCA cycle, fatty acid and phospholipid metabolism and PHB synthesis (Fig. 1). We observed that the co-overexpression of *plsC/plsX* could overcome the limitation of lipid production after treating with acetate up to 39.1%w/dcw for 4 days of treatment while the gradual decreases was observed at 6 and 8 days of treatment. To partially understand the transcriptional regulation under acetate treatment, our results suggested that acetate addition itself highly induced gene transcript levels of WT related to phospholipid and PHB syntheses, and decreased lipid hydrolysis via LipA. For the co-overexpresion of *plsC/plsX*, results indicated that acetate was capable of decreasing *phaA* transcript levels, which involved in PHB synthesis, and redirecting acetyl-CoA into fatty acid and phospholipid metabolism. Moreover, this study demonstrated the high level of lipid production when compared to some other modified microorganisms in different strategies (shown in Table 4). The sustainability aspect for the third generation of biofuel resource might be effectively archived by genetic engineering approach, such as the genetic modified cyanobacteria with continuously secreting fatty acid production.

## Methods

### Organisms used in this study and growth condition

*Escherichia coli* strain DH5α was used as a host for plasmid propagation. *Synechocystis* sp. PCC 6803 wild type cells, control WT strain (wild type cell containing empty pEERM vector) and both overexpressing strains of *plsX, plsC* and *plsC/plsX* (Table 2) were cultivated in liquid BG_11_ medium at 30 °C under continuous light intensity of 50 µmol photons m^−2^ s^−1^ for 20 days. Cell growth was monitored by a measurement of optical density (OD) at 730 nm using a spectrophotometer. For the overexpressing strains, they were grown in BG11 medium with the presence of antibiotic chloramphenicol (30 µg.mL^−1^). For nutrient modified treatment, the cell culture with mid-logarithmic phase of growth was harvested by centrifuging at 6,000 rpm (4,025 × g), 25 °C for 10 min and transferred cell pellets to modified BG_11_ media using unmodified BG_11_ medium as a control. Modified BG_11_ medium consisted of BG_11_ medium supplemented with 0.4% (w/v, 6.7 mM) acetate.

**Table 2:** Strains and plasmids used.

Name	Relevant genotype	Reference
**Cyanobacterial strains**		
*Synechocystis* PCC 6803	Wild type	Pasteur Culture Collection
OX *plsX*	*plsX*, *Cm*^*r*^ integrated at flanking region of *psbA2* gene in *Synechocystis* genome	This study
OX *plsC*	*plsC*, *Cm*^*r*^ integrated at flanking region of *psbA2* gene in *Synechocystis* genome	This study
OX *plsC/plsX*	*plsC/plsX*, *Cm*^*r*^ integrated at flanking region of *psbA2* gene in *Synechocystis* genome	This study
WT control	WT, *Cm*^*r*^ integrated at flanking region of *psbA2* gene in *Synechocystis* genome	This study
**Plasmids**		
pEERM PCC 6803	P_psbA2_- *Cm*^*r*^; plasmid containing flanking region of *psbA2* gene	^17^
pEERM_*plsC*	P_psbA2_-*plsC*-*Cm*^*r*^; integrated between XbaI and SpeI sites of pEERM	This study
pEERM_*plsX*	P_psbA2_-*plsX*-*Cm*^*r*^; integrated between SpeI and PstI sites of pEERM	This study
pEERM_*plsC/plsX*	P_psbA2_-*plsX*-*Cm*^*r*^; integrated between SpeI and PstI sites of pEERM_*plsC*	This study

P_psbA2_, strong *psbA2* promoter; *Cm*^*r*^, chloramphenicol antibiotic resistance cassette.

### The construction and transformation of overexpressing Synechocystis strains

*Synechocystis* sp. PCC 6803 genomic DNA prepared was used as the DNA template for amplifying *sll1848* gene fragment. The *plsC* (or *sll1848*) and *plsX* (or *slr1510*) gene fragments were amplified by PCR method using each specific pair of primers (Table 3). PCR was performed using an initial denaturation at 98 °C for 30 sec, followed by 29 cycles for *plsC* and 26 cycles for *plsX* of three steps including denaturation at 94 °C for 10 sec, annealing step of each specific pair of primers at 55 °C for 30 sec and extension at 72 °C for 25 sec, followed by final extension at 72 °C for 5 min. The PCR products were then checked by 0.8% gel electrophoresis using 1xTAE buffer. After PCR amplification, those gene fragments was digested with specific restriction enzymes (Table 2) and further cloned into the expression vector pEERM^17^ using T4 DNA ligase. These obtained recombinant plasmids (Table 2) was then transformed into *Escherichia coli* DH5-α strain using calcium chloride method. Cells were spread on LB agar containing 30 µg.mL^−1^ chloramphenicol and checked by restriction enzyme digestion and agarose gel electrophoresis.

**Table 3:** Primers used in this study.

Target gene	Primer	Sequence (5′$\to$ 3′)	Product size (bp)
*PlsC*	Forward	CTAGTCTAGAGTGGATTCCGAGATTAAT	678
*PlsC*	Reverse	CTAGACTAGTCTAATCCCTGCCTAAATCCAGCAT	
*PlsX*	Forward	TAGAGAACTAGTATGGCTGTAACGCGG	1,047
*PlsX*	Reverse	TAGAGACTGCAGCTAGATATTCTGTAATTCCTC	
*Cm* ^*r*^	Forward	GAGTTGATCGGGCACGTAAG	899
*Cm* ^*r*^	Reverse	CTCGAGGCTTGGATTCTCAC	
*UpsbA2*	Forward	TGCCTGTCAGCAAAACAACTT	2,841
*DpsbA2*	Reverse	CGAGGGCAATCATCAATTCCG	
*16* *s*	Forward	AGTTCTGACGGTACCTGATGA	521(RT-PCR)
*16s*	Reverse	GTCAAGCCTTGGTAAGGTTAT	
*PlsC*	Forward	TCTCTACCGGGGCTTGAAATG	508(RT-PCR)
*PlsC*	Reverse	CGCCTTACCAATGCGAATAGT	
*PlsX*	Forward	AAGGGGTGGTGGAAATGGAA	488(RT-PCR)
*PlsX*	Reverse	AAGTAGGTCCCTTCCTTCGG	
*AccA*	Forward	ATGCACGGCGATCGAGGAGGT	428(RT-PCR)
*AccA*	Reverse	TGGAGTAGCCACGGTGTACAC	
*Aas*	Forward	CCCATTGAAGATGCCTGTTT	304(RT-PCR)
*Aas*	Reverse	GTGCTGGGATAAAACGGAAA	
*PhaA*	Forward	CATGATGGTTTGACGGACAG	310(RT-PCR)
*PhaA*	Reverse	GACTACAGTTGCCCGCTGTT	
*LipA*	Forward	TTGGCGGAGCAAGTGAAGCAAT	379(RT-PCR)
*LipA*	Reverse	ATTTTGCCTGTGCTGGTCCATG

**Table 4:** Lipid production in some engineered microorganism strains by different experimental designs.

Microorganism strains	Engineered design	Lipid content	Condition used	Ref.
**Enhancement of intracellular lipid content**				
Bacterium *Escherichia coli*	By overexpression of genes encoding four subunits of native ACC under the control of bacteriophage T7 promoter.	6.69 nmol (6 fold increase)	The medium was rich broth (per liter; 10 g of tryptone, 1 g of yeast extract, 5 g of NaCl), grown at 37 ºC.	^27^
Yeast *Saccharomyces cerevisiae*	By site-directed mutagenesis of S659 and A1157 to reduce SNF1-mediated phosphorylation of *Acc1*; overexpression of *Acc1* WT and mutants.	11.7 ± 2.0%w/CDW (65% increase)	Culture was in a synthetic medium with 20 g/liter glucose with the controlled temperature of 30 °C.	^34^
Green algae *Chlamydomonas reinhardtii*	By genetically engineering with a key enzyme diacylglycerol acyltransferase (*BnDGAT2*) from *Brassica napus*.	18.76%w/CDW (1.5 fold increase)	Culture in TAP medium at 25 °C, with light intensity 12000 lux (for 16: 8 h light and dark condition) on solid plates or shaken at 200 rpm.	^35^
Green algae *Chlamydomonas reinhardtii*	By overexpressing three putative type-2 DGAT2 candidate genes.	28–36%w/CDW	Culture in various TAP media; TAP, TAP-N (no nitrogen), TAP-S (no sulfur) under continuous white light (40 μE/m^2^/s)	^36^
Cyanobacterium *Synechocystis* sp. PCC 6803	By overexpressing native *PlsX, PlsC, PlsX/PlsC* genes via pEERM expression vector	24.3%w/dcw(*PlsX/PlsC* strain)	Culture in BG-11 medium (normal condition), at 30 °C under continuous light intensity of 50 µE/m^2^/s	This study
		39.1%w/dcw (*PlsX/PlsC* strain)	Culture in BG-11 medium with acetate supplementation, at 30 °C under continuous light intensity of 50 µE/m^2^/s	This study
**Enhancement of lipid (or free fatty acid) secretion into medium**				
Cyanobacterium *Synechocystis* sp. PCC 6803	By adding codon-optimized thioesterase genes and weakening polar cell wall layers.	Fatty acid secretion 197 ± 14 mg/L (1.97%w/v)	A cell density of 1.0 × 10^9^ cells/mL grown in BG-11 medium with 100 mL∕ min aeration of 1% CO_2_-enriched air.	^37^
Cyanobacterium *Synechococcus sp. PCC 7002*	By overexpression of non-native RuBisCO subunits (*rbcLS*) with gene knockout of theacyl-ACPsynthetase/long-chain-fatty-acid CoA ligase (*fadD*),	Fatty acid secretion 131 mg/L (1.31%w/v)	Cells in medium A+ with antibiotic supplementation grown at 30 or 38 °C bubbling of 1% CO_2_ in air.	^38^
Cyanobacterium *Synechococcus elongates* PCC 7942	By gene knockout of the FFA-recycling acyl-ACP synthetase and expression of a thioesterase for release of the FFA.	Fatty acid secretion 49.3 mg/L (0.493%w/v)	Cells in medium BG-11 with antibiotic supplementation grown at 30 °C bubbling of filter-sterilized air supplemented with 1% CO_2_.	^39^

For transformation of recombinant plasmids into cells of *Synechocystis*, the recombinant plasmids and empty vector were independently transformed into *Synechocystis* cells by natural transformation method. Ten μL of the recombinant plasmid solution was added into the tube and incubated under normal growth light condition at 30 °C for 6 hours. Next, a reaction mixture was spread on BG_11_ agar containing 10 μg/ml chloramphenicol. Incubation at 30 °C for 2–3 weeks was performed until single green colony was appeared. Those cell tranformants were selected on BG_11_ agar containing higher concentration of chloramphenicol up to 30 μg.mL^−1^. After that, the obtained transformant was used as a template for checking both size and gene location by PCR method with different pairs of primers (Table 3).

### Determinations of pigment contents and oxygen evolution rate

The contents of chlorophyll *a* and carotenoids of *Synechocystis* were extracted by N, N-dimethylformamide (DMF) method. One ml of cell culture was harvested by centrifuging at 10,000 rpm (17,507 × g) at 25 °C for 10 min and discarded supernatant. The obtained pellet was further carried out by extracting with N, N-dimethylformamide (DMF) and incubated under darkness for 10 min. Then, centrifugation at 10,000 rpm (17,507 × g) 25 °C for 10 min was performed. Later, the supernatant was measured its absorbances at 461, 625, and 664 nm, respectively. The pigment contents were calculatedaccording to^30^ and^31^ equations.

A cell culture (5 mL) was harvested by centrifuging at 8000 × g for 10 min and cell pellets were resuspended in 2 ml of fresh BG_11_ medium. The incubation under darkness about 30 min was performed before measuring oxygen evolution by Oxygraph plus oxygen electrode (Hansatech Instruments, U.K.). The oxygen evolution measurement was done at 25 °C using fluorescent light as a saturated light source. The unit of oxygen evolution rate is presented as μmol O_2_.mg Chl *a*^−^1.h^−1^.

### Determinations of total lipid and unsaturated lipid contents

The complete cell was measured for total lipid content by the dichromate oxidation method^32^. Standard lipid stock was prepared using commercial canola oil. *Synechocystis* cell culture (5 mL) was harvested by centrifuging at 8000 × g for 10 min. The cell pellet was further added 2 mL of concentrated sulfuric acid (98%) and 2 mL of potassium dichromate solution, and boiled that mixture for 30 min at 100 °C, followed by cooling for 10 min on ice bath. After the incubation at room temperature for 10 min, two mL of distilled water was added and mixed. The sample was measured its absorbance at 600 nm by spectrophotometer. Unit of total lipid content was %w/w of dry cell weight (dcw). The dry cell weight was performed by incubating at 80 °C for 48 hours until reaching the constant dry cell weight.

The unsaturated lipid content was measured by colorimetric sulfo-phospho-vanillin (SPV) method^33^. The standard lipid stock was prepared using commercial gamma-linolenic acid. Cell culture (5 mL) for lipid quantification was harvested by centrifugation at 8000 × g for 10 min. The 2 mL of concentrated sulfuric acid (98%) was added into the sample before boiling for 30 min at 100 °C and cooling later for 10 min on ice bath. Two mL of freshly prepared phospho-vanillin reagent was then added. The sample mixture was then incubated for 10 min at room temperature. After that, an absorbance reading at 540 nm was performed in order to measure the unsaturated lipid content. Unit of total unsaturated lipid content was %w/w of dry cell weight (dcw).

### Analysis of fatty acid composition

Cell culture (1 L) with optical density about 0.8, was harvested by centrifuging at 6,000 rpm (4,025 × g), 25 °C, for 10 min. Harvested samples were hydrolyzed and further derivatized into the corresponding fatty acid methyl esters (FAMEs). FAMEs was determined using a GC-MS (model) instrument.

### Reverse transcription-polymerase chain reaction (RT-PCR)

The isolation of total RNA was performed from harvested cells using Trizol® Reagent (Invitrogen, USA). The first stand cDNA synthesis was used in one microgram of total isolated RNA. The reaction was performed by SuperScriptTM III First-Strand Synthesis System kit (Invitrogen, USA). RT-PCR amplifications using cDNAs of the respective genes were performed using corresponding primers listed in Table 3. The PCR reaction was consisted of initial denaturation at 98 °C for 30 sec, followed by by 29 cycles for *PlsC* and other genes studied, and 14 and 26 cycles for 16* s* and *PlsX*, respectively, of three steps including denaturation at 94 °C for 10 sec, annealing step of each specific pair of primers at 55 °C for 30 sec and extension at 72 °C for 25 sec, followed by final extension at 72 °C for 5 min. The PCR products were analyzed by 0.8% (w/v) agarose gel electrophoresis and quantification was done using Syngene® Gel Documentation (Syngene, Frederick, MD).

## Electronic supplementary material

Supplementary information

## Data Availability

All data generated or analysed during this study are included in this article.

## References

[1] Quintana N, Van der Kooy F, Van de Rhee M, Voshol GP, Verpoorte R (2011). Renewable Energy from Cyanobacteria: Energy Production Optimization by Metabolic Pathway Engineering. Applied Microbiology and Biotechnology.

[2] Heyer H, Krumbein WE (1991). Excretion of Fermentation Products in Dark and Anaerobically Incubated Cyanobacteria. Archives of Microbiology.

[3] Winters K, Parker PL, Baalen CV (1969). Hydrocarbons of Blue-Green Algae: Geochemical Significance. Science.

[4] Karatay SE, Dömmez G (2011). Microbial Oil Production from Thermophile Cyanobacteria for Biodiesel Production. Applied Energy.

[5] Gao X, Sun T, Wu L, Chen L, Zhang W (2017). Co-overexpression of Response Regulator Genesslr1037 and sll0039 Improves Tolerance of Synechocystis sp. PCC 6803 to 1-Butanol. Bioresource Technology.

[6] Yao L, Qi F, Tan X, Lu X (2014). Improved Production of Fatty Alcohols in Cyanobacteria by Metabolic Engineering. Biotechnology for Biofuels.

[7] Shestakov SV, Mikheev LE (2006). Genetic Control of Hydrogen Metabolism in Cyanobacteria. Russian Journal of Genetics.

[8] Baebprasert W, Jantaro S, Khetkorn W, Lindblad P, Incharoensakdi A (2011). Increased H_2_ Production in the Cyanobacterium *Synechocystis* sp. Strain PCC 6803 by Redirecting the Electron Supply via Genetic Engineering of the Nitrate Assimilation Pathway. Metabolic Engineering.

[9] Sakamoto T, Wada H, Nishida I, Ohmori M, Murata N (1994). Delta 9 Acyl-Lipid Desaturases of Cyanobacteria. Molecular Cloning and Substrate Specificities in Terms of Fatty Acids, sn-Positions, and Polar Head Groups. The Journal of Biological Chemistry.

[10] Kaczmarzyk D, Fulda M (2010). Fatty Acid Activation in Cyanobacteria Mediated by Acyl-Acyl Carrier Protein Synthetase Enables Fatty Acid Recycling. Plant Physiology.

[11] Weier D, Müller C, Gaspers C, Frentzen M (2005). Characterisation of Acyltransferases from *Synechocystis* sp. PCC 6803. Biochemical and Biophysical Research Communications.

[12] Okazaki K, Sato N, Tsuji N, Tsuzuki M, Nishida I (2006). The Significance of C16 Fatty Acids in the sn-2 Positions of Glycerolipids in the Photosynthetic Growth of *Synechocystis* sp. PCC 6803. Plant Physiology.

[13] Yu B, Wakao S, Fan J, Benning C (2004). Loss of Plastidic Lysophosphatidic Acid Acyltransferase Causes Embryo-Lethality in *Arabidopsis*. Plant and Cell Physiology.

[14] Zhang YM, Rock CO (2008). Thematic Review Series: Glycerolipids. Acyltransferases in Bacterial Glycerophospholipid Synthesis. Journal of Lipid Research.

[15] Lu YJ (2006). Acyl-Phosphates Initiate Membrane Phospholipid Synthesis in Gram-Positive Pathogens. Molecular Cell.

[16] Paoletti L, Lu YJ, Schujman GE, de Mendoza D, Rock CO (2007). Coupling of Fatty Acid and Phospholipid Synthesis in *Bacillus subtilis*. Journal of Bacteriology.

[17] Englund E, Andersen-Ranberg J, Miao R, Hamberger B, Lindberg P (2015). Metabolic Engineering of *Synechocystis* sp. PCC 6803 for Production of the Plant Diterpenoid Manoyl Oxide. *ACS Synthetic*. Biology.

[18] Khetkorn W, Incharoensakdi A, Lindblad P, Jantaro S (2016). Enhancement of Poly-3-Hydroxybutyrate Production in *Synechocystis* sp. PCC 6803 by Overexpression of Its Native Biosynthetic Genes. Bioresource Technology.

[19] Mohamed A, Jansson C (1989). Influence of Light on Accumulation of Photosynthesis-Specific Transcripts in the Cyanobacterium *Synechocystis* 6803. Plant Molecular Biology.

[20] Mohamed A, Eriksson J, Osiewacz HD, Jansson C (1993). Differential Expression of the *psbA* Genes in the Cyanobacterium *Synechocystis* 6803. Molecular Genetics and Genomics.

[21] Van Ginneken VJT, Helsper JPFG, de Visser W, Van Keulen H, Brandenburg WA (2011). Polyunsaturated Fatty Acids in Various Macroalgal Species from North Atlantic and Tropical Seas. Lipids in Health and Disease.

[22] De Mendoza D, Cronan JE (1983). Thermal Regulation of Membrane Lipid Fluidity in Bacteria. Trends in Biochemical Sciences.

[23] Wada H, Murata N (1990). Temperature-Induced Changes in the Fatty Acid Composition of the Cyanobacterium *Synechocystis* PCC6803. Plant Physiology.

[24] Morgan-Kiss RM, Cronan JE (2008). The *Lactococcus lactis* FabF Fatty Acid Synthetic Enzyme Can Functionally Replace Both the FabB and FabF Proteins of *Escherichia coli* and the FabH Protein of *Lactococcus lactis*. Archives of Microbiology.

[25] Edwards P, Sabo Nelsen J, Metz JG, Dehesh K (1997). Cloning of the *fabF* Gene in an Expression Vector and *in vitro* Characterization of Recombinant *fabF* and *fabB* Encoded Enzymes from *Escherichia coli*. FEBS Letters.

[26] Fujita Y, Matsuoka H, Hirooka K (2007). Regulation of Fatty Acid Metabolism in Bacteria. Molecular Microbiology.

[27] Davis MS, Solbiati J, Cronan JE (2000). Overproduction of acetyl-CoA carboxylase activity increases the rate of fatty acid biosynthesis in *Escherchia coli*. The Journal of Biological Chemistry.

[28] Martin SA, Gijόn MA, Voelker DR, Murphy RC (2014). Measurement of lysophospholipid acyltransferase activities using substrate competition. Journal of Lipid Research.

[29] Yang W (2010). A distinct type of glycerol-3-phosphate acyltransferase with sn-2 preference and phosphatase activity producing 2-monoacylglycerol. PNAS.

[30] Moran R (1982). Formulae for Determination of Chlorophyllous Pigments Extracted with N, N-Dimethylformamide. Plant Physiology.

[31] Chamovitz D, Sandmann G, Hirschberg J (1993). Molecular and Biochemical Characterization of Herbicide-Resistant Mutants of Cyanobacteria Reveals That Phytoene Desaturation is a Rate-Limiting Step in Carotenoid Biosynthesis. The Journal of Biological Chemistry.

[32] Fales FW (1971). Evaluation of a Spectrophotometric Method for Determination of Total Fecal Lipid. Clinical Chemistry.

[33] Cheng YS, Zheng Y, Vander Gheynst JS (2011). Rapid Quantitative Analysis of Lipids Using a Colorimetric Method in a Microplate Format. Lipids.

[34] Shi S, Chen Y, Siewers V, Nielsen J (2014). Improving production of malonyl coenzyme A-derived metabolites by abolishing Snf1-dependent regulation of Acc1. American Society for Microbiology.

[35] Ahmad I, Sharma AK, Daniell H, Kumar S (2015). Altered lipid composition and enhanced lipid production in green microalga by introduction of brassica diacylglycerol acyltransferase 2. Plant Biotechnology Journal.

[36] La Russa M (2012). Functional analysis of three type-2 DGAT homologue genes for triacylglycerol production in the green microalga *Chlamydomonas reinhardtii*. Journal of Biotechnology.

[37] Liu X, Sheng J, Curtiss R (2011). Fatty acid production in genetically modified cyanobacteria. PNAS.

[38] Ruffing AM (2014). Improved free fatty acid production in cyanobacteria with *Synechococcus* sp. PCC 7002 as host. Frontiers in Bioengineering and Biotechnology.

[39] Ruffing AM, Jones HDT (2012). Physiological effects of free fatty acid production in genetically engineered *Synechococcus elongatus* PCC 7942. Biotechnology and Bioengineering.

