# Supplementary material for: Co-overexpression of native phospholipid-biosynthetic genes plsX and plsC enhances lipid production in Synechocystis sp. PCC 6803
Source: Sci Rep. 2018 Sep 10;8:13510. doi: 10.1038/s41598-018-31789-5 (PMC6131169; doi:10.1038/s41598-018-31789-5)
Supplement: Supplementary file 1 — Supplementary information [file 41598_2018_31789_MOESM1_ESM.pdf]

Co-overexpression of native phospholipid-biosynthetic genes *plsX* and *plsC* enhances lipid production in *Synechocystis* sp. PCC 6803

Umaporn Towijit, Nutchaya Songruk, Peter Lindblad, Aran Incharoensakdi & Saowarath Jantaro*

Supplementary Figure S1

Phylogenetic tree analysis. The amino acid sequence of fatty acid/phospholipid synthesis protein PlsX in 22 cyanobacterial strains from cyanobase and other known species from NCBI database (Table S1)

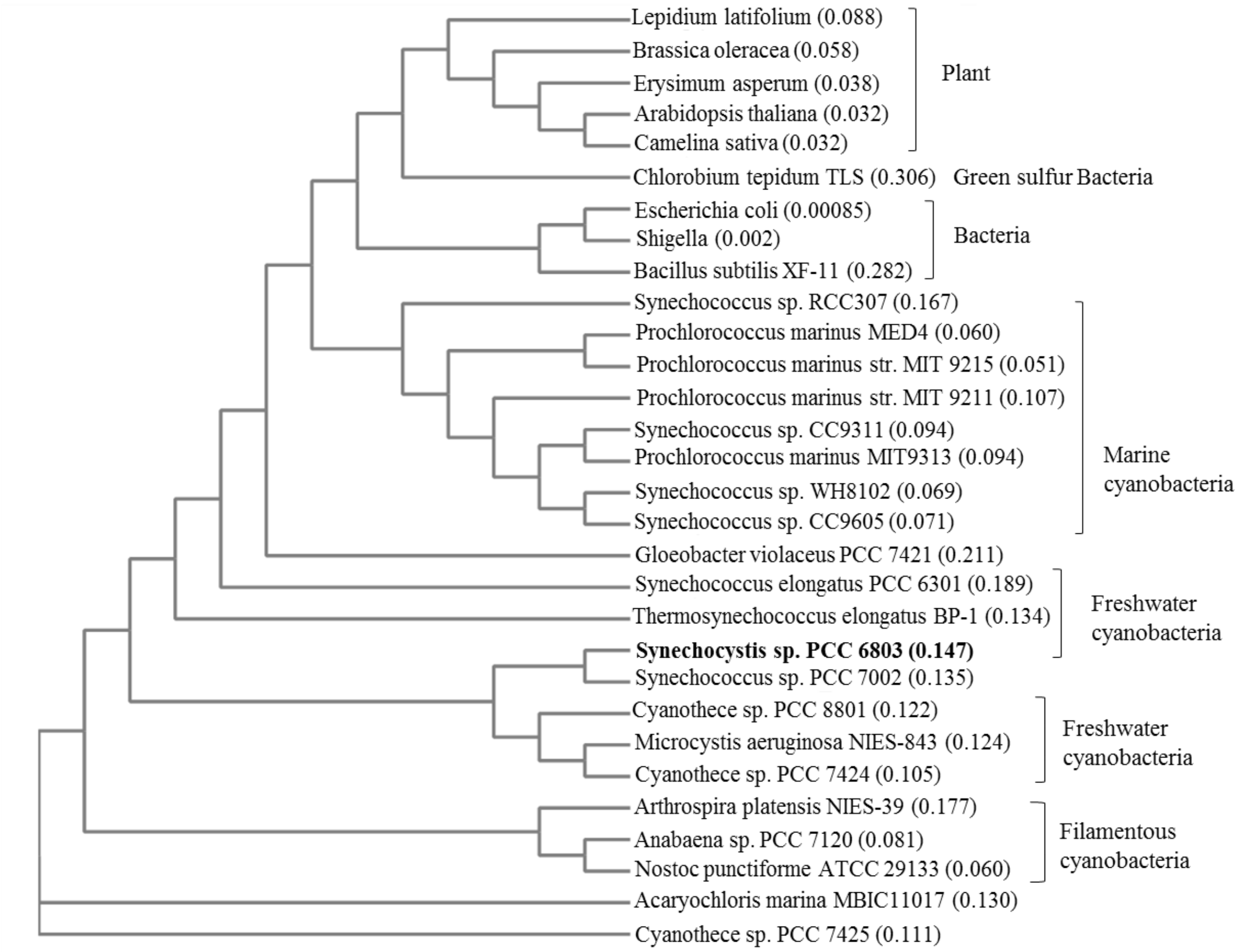

Supplementary Table S1 PlsX: Gene codes, gene indexing names, organisms and sources in cyanobacteria and other species

Gene code	Gene indexing name	Organisms	Source
**Cyanobacteria**			Cyanobase
Slr1510	Fatty acid/phospholipid synthesis protein PlsX	*Synechocystis* sp. PCC 6803	
Alr0238	Fatty acid/phospholipid synthesis protein PlsX	*Anabaena* sp. PCC 7120	
Tlr0844	Fatty acid/phospholipid synthesis protein PlsX	*Thermosynechococcus elongatus* BP-1	
Gll0800	Fatty acid/phospholipid synthesis protein PlsX	*Gloeobacter violaceus* PCC 7421	
MAE19480	Fatty acid/phospholipid synthesis protein PlsX	*Microcystis aeruginosa* NIES-843	
PMM0135	Fatty acid/phospholipid synthesis protein PlsX	*Prochlorococcus marinus* MED4	
PMT1995	Fatty acid/phospholipid synthesis protein PlsX	*Prochlorococcus marinus* MIT9313	
SYNW2247	Fatty acid/phospholipid synthesis protein PlsX	*Synechococcus* sp. WH8102	
syc0103_c	Fatty acid/phospholipid synthesis protein PlsX	*Synechococcus elongatus* PCC 6301	
sync_2598	Fatty acid/phospholipid synthesis protein PlsX	*Synechococcus* sp. CC9311	
SYNPCC7002_A0526	Fatty acid/phospholipid synthesis protein PlsX	*Synechococcus* sp. PCC 7002	
SynRCC307_0249	Fatty acid/phospholipid synthesis protein PlsX	*Synechococcus* sp. RCC307	
Syncc9605_2385	Fatty acid/phospholipid synthesis protein PlsX	*Synechococcus* sp. CC9605	
AM1_0652	Fatty acid/phospholipid synthesis protein PlsX	*Acaryochloris marina* MBIC11017	
P9215_01521	Fatty acid/phospholipid synthesis protein PlsX	*Prochlorococcus marinus* str. MIT 9215	
P9211_01491	Fatty acid/phospholipid synthesis protein PlsX	*Prochlorococcus marinus* str. MIT 9211	
Npun_F0098	Fatty acid/phospholipid synthesis protein PlsX	*Nostoc punctiforme* ATCC 29133	
PCC7424	Fatty acid/phospholipid synthesis protein PlsX	*Cyanothece* sp. PCC 7424	
Cyan7425_2501	Fatty acid/phospholipid synthesis protein PlsX	*Cyanothece* sp. PCC 7425	
PCC8801_1806	Fatty acid/phospholipid synthesis protein PlsX	*Cyanothece* sp. PCC 8801	
NIES39_N01180	Fatty acid/phospholipid synthesis protein PlsX	*Arthrospira platensis* NIES-39	
**Bacteria**			NCBI
gi|585334175	phosphate acyltransferase	*Escherichia coli*	
gi|585377077	phosphate acyltransferase plsX	*Shigella*	
gi|449028196	phosphate acyltransferase plsX	*Bacillus subtilis* XF-11	
CT2113	Fatty acid/phospholipid synthesis protein PlsX	*Chlorobium tepidum* TLS	
**Plant**			
gi|332193327	glycerol-3-phosphate acyltransferase	*Arabidopsis thaliana*	
gi|663499089	glycerol-3-phosphate acyltransferase	*Erysimum asperum*	
gi|347449184	glycerol-3-phosphate acyltransferase	*Lepidium latifolium*	
gi|727436894	glycerol-3-phosphate acyltransferase	*Camelina sativa*	
gi|922544539	glycerol-3-phosphate acyltransferase	*Brassica oleracea*	

Supplementary Figure S2

Unrooted phylogenetic tree analysis of the amino acid sequences of 1-acyl-sn-glycerol-3-phosphate acyltransferase from 28 strains of cyanobacteria, and other known species (Table S2)

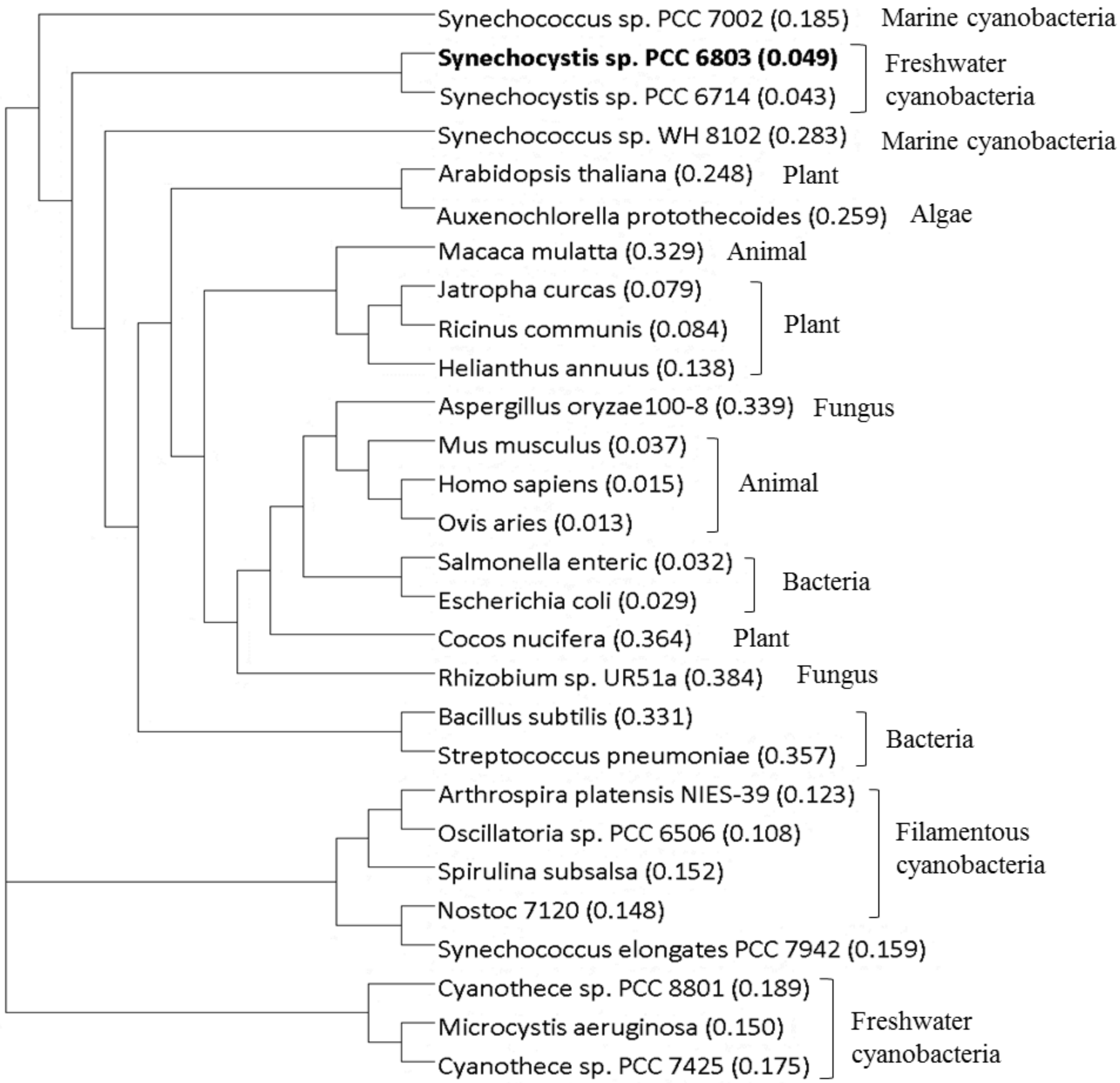

Table S2 PlsC: Gene codes, gene indexing names, organisms and sources in cyanobacteria and other species

Gene codes	Gene indexing name	Organisms	Sources
**Cyanobacteria**			
WP_010873224.1	1-acyl-sn-glycerol-3-phosphate acyltransferase	*Synechocystis* sp. PCC 6803	Cyanobase
WP_051738848.1	1-acyl-sn-glycerol-3-phosphate acyltransferase	*Synechocystis* sp. PCC 6714	NCBI
ACL46701.1	1-acyl-sn-glycerol-3-phosphate acyltransferase	*Cyanothece* sp. PCC 7425	Cyanobase
ACK65339.1	1-acyl-sn-glycerol-3-phosphate acyltransferase	*Cyanothece* sp. PCC 8801	NCBI
WP_002792013.1	1-acyl-sn-glycerol-3-phosphate acyltransferase	*Microcystis aeruginosa*	NCBI
ABB57487.1	1-acyl-sn-glycerol-3-phosphate acyltransferase	*Synechococcus elongates* PCC 7942	Cyanobase
ACB00374.1	1-acyl-sn-glycerol-3-phosphate acyltransferase	*Synechococcus sp.* PCC 7002	Cyanobase
NP_898339	1-acyl-sn-glycerol-3-phosphate acyltransferase	*Synechococcus* sp. WH 8102	NCBI
WP_026080032.1	1-acyl-sn-glycerol-3-phosphate acyltransferase	*Spirulina subsalsa*	NCBI
CBN57772.1	1-acyl-sn-glycerol-3-phosphate acyltransferase	*Oscillatoria* sp. PCC 6506	NCBI
BAI94386.1	1-acyl-sn-glycerol-3-phosphate acyltransferase	*Arthrospira platensis* NIES-39	NCBI
NP_484285	1-acyl-sn-glycerol-3-phosphate acyltransferase	*Nostoc* sp. PCC 7120	NCBI
**Algae**			
KFM23593.1	1-acyl-sn-glycerol-3-phosphate acyltransferase	*Auxenochlorella protothecoides*	NCBI
**Bacteria**			
Q8DNY1.1	1-acyl-sn-glycerol-3-phosphate acyltransferase	*Streptococcus pneumonia*	NCBI
BAM49875.1	1-acyl-sn-glycerol-3-phosphate acyltransferase	*Bacillus subtilis*	
P0A257.1	1-acyl-sn-glycerol-3-phosphate acyltransferase	*Salmonella enteric*	
P26647.1	1-acyl-sn-glycerol-3-phosphate acyltransferase	*Escherichia coli*	
**Fungus**			
KDE82468.1	1-acyl-sn-glycerol-3-phosphate acyltransferase	*Aspergillus oryzae*100-8	NCBI
WP_004439605.1	1-acyl-sn-glycerol-3-phosphate acyltransferase	*Rhizobium* sp. UR51a	
**Plants**			
Q8GXU8.1	1-acyl-sn-glycerol-3-phosphate acyltransferase	*Arabidopsis thaliana*	NCBI
ABU50327.1	1-acyl-sn-glycerol-3-phosphate acyltransferase	*Helianthus annuus*	
ACC59198.1	1-acyl-sn-glycerol-3-phosphate acyltransferase	*Ricinus communis*	
NP_001295696.1	1-acyl-sn-glycerol-3-phosphate acyltransferase	*Jatropha curcas*	
Q42670.1	1-acyl-sn-glycerol-3-phosphate acyltransferase	*Cocos nucifera*	
**Animals**			
O35083.1	1-acyl-sn-glycerol-3-phosphate acyltransferase	*Mus musculus*	NCBI
Q99943.2	1-acyl-sn-glycerol-3-phosphate acyltransferase	*Homo sapiens*	
Q95JH0.1	1-acyl-sn-glycerol-3-phosphate acyltransferase	*Ovis aries*	
AFE79273.1	1-acyl-sn-glycerol-3-phosphate acyltransferase	*Macaca mulatta*	
